# Deoxynivalenol Inhibits Porcine Intestinal Trefoil Factors Expression in Weanling Piglets and IPEC-J2 Cells

**DOI:** 10.3390/toxins11110670

**Published:** 2019-11-15

**Authors:** Shuai Wang, Cong Zhang, Xu Wang, Jiacheng Yang, Kuntan Wu, Jiacai Zhang, Beiyu Zhang, Ao Yang, Desheng Qi

**Affiliations:** Department of Animal Nutrition and Feed Science, College of Animal Science and Technology, Huazhong Agricultural University, Wuhan 430070, China; wangshuai@mail.hzau.edu.cn (S.W.); zhangcong@webmail.hzau.edu.cn (C.Z.); xuwang@webmail.hzau.edu.cn (X.W.); yangjiacheng@webmail.hzau.edu.cn (J.Y.); kuntanwu@webmail.hzau.edu.cn (K.W.); zjc404@webmail.hzau.edu.cn (J.Z.); zhangbeiyu@webmail.hzau.edu.cn (B.Z.); yangao@webmail.hzau.edu.cn (A.Y.)

**Keywords:** deoxynivalenol, intestine, trefoil factors, SPDEF, weanling piglet

## Abstract

Trefoil factors (TFFs) are regulatory peptides playing critical roles in mucosal repair and protection against a variety of insults within the gastrointestinal tract. This work aimed to explore the effects of deoxynivalenol (DON) on intestinal TFFs expression using in vivo and in vitro models. In an animal trial, twenty-four 28-d-old barrows (Duroc × Landrace × Large White; initial body weight = 7.6 ± 0.7 kg) were randomly divided into three treatments for 28 days, including a control diet (0.61 mg DON/kg feed), and two levels of DON-contaminated diets containing 1.28 and 2.89 mg DON/kg feed, respectively. Piglets exposed to DON had lower mRNA expression of TFF1, TFF2, TFF3, as well as Claudin-4 in the intestine (*P* < 0.05). Dietary DON exposure decreased the protein levels of TFF2 and TFF3 in the jejunum as demonstrated by western blot and immunohistochemistry. In intestinal porcine epithelial cells (IPEC-J2), DON depressed the mRNA expression of TFF2, TFF3, and Claudin-4. Overexpression of sterile alpha motif (SAM) pointed domain E26 transformation-specific (ETS) factor (SPDEF) was found to attenuate DON-induced suppression of TFFs in IPEC-J2 cells. Altogether, our work shows, for the first time, that dietary DON exposure depresses the expression of intestinal TFFs in piglets. Given the fundamental role of TFFs in intestinal mucosal homeostasis, our observations indicate that the DON content in animal feed should be strictly controlled based on the existing regulation for DON.

## 1. Introduction

Mycotoxins are fungal secondary metabolites frequently occurring in the food chain and posing risk to human and animal health [1]. Among mycotoxins, deoxynivalenol (DON) is one of the most pervasive mycotoxins contaminating cereals as well as animal feeds and finished food. DON is toxic to various animal species and even humans, with swine being the highest sensitive species [2]. The intestine is the initial line of defense against food contaminants and the primary target for DON [3]. A recent study has demonstrated that DON intoxicated intestinal health by depressing the expression of trefoil factors (TFFs) in goblet cells [4]. DON induced inhibition of TFFs contributes to impaired intestinal integrity through alteration of mucosal regeneration or repair [4]. Trefoil factors (TFF1, 2, and 3) are a family of protease-resistant peptides that are abundantly secreted onto the surface of the intestine by mucin-producing cells. They play a fundamental role in mucosal repair and protection against various insults in the gastrointestinal tract [5]. Disturbances of TFFs production have been observed in patients with chronic enteric diseases, such as Crohn’s disease and ulcerative colitis [6,7]. 

At the intestinal level, DON was shown to deteriorate intestinal structure, disrupt barrier function, reduce nutrient absorption, as well as alter intestinal immunity [8,9,10]. However, in piglets, the effect of DON on the intestinal TFFs expression and its molecular mechanism has not been investigated to date. Sterile alpha motif (SAM) pointed domain E26 transformation-specific (ETS) factor (SPDEF) is an ETS family transcription factor involved in regulating intestinal epithelial cell differentiation and homeostasis. Growing evidence suggests SPDEF is indispensable for the production of TFFs [11,12]. SPDEF mutations and depressed TFFs expression have been linked to an accumulation of immature Paneth and goblet cells in the intestine [13]. 

The purpose of this work was to explore the effects of DON on the intestinal expression of TFFs using in vivo and in vitro models. In vivo experiments were carried out to determine the effects on abundance and localization of TFFs in the intestine of piglets exposed to 1.28 and 2.89 mg DON/kg feed. In vitro, a gene overexpression technique was used to identify the role of SPDEF in alleviating DON-induced dysregulation of TFFs using IPEC-J2 cells.

## 2. Results

### 2.1. mRNA Expressions of TFFs Genes in the Intestine

**Figure 1:**
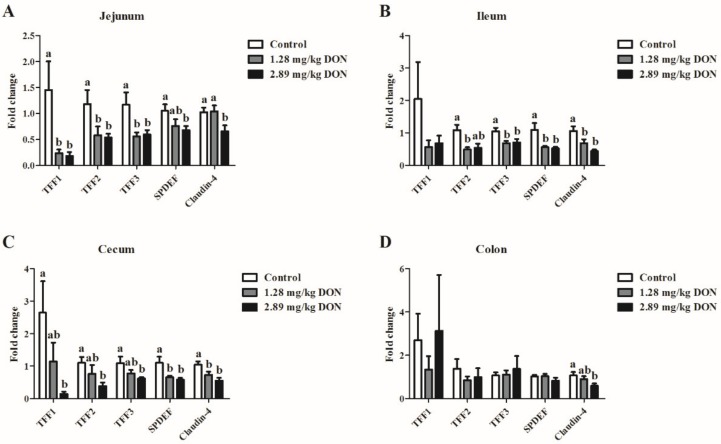
Effect of dietary deoxynivalenol (DON) exposure on the mRNA expression of trefoil factors and claudin-4 in the jejunum (**A**), ileum (**B**), cecum (**C**), and colon (**D**). Piglets were fed a control diet (□) or a diet contaminated with 1.28 (■) and 2.89 mg DON/kg feed (■). Values are means ± SEM, n = 8. ^a,b^ Mean values without a common letter differ (*P* < 0.05). SEM, standard error of mean.

As shown in Figure 1, the relative abundances of TFF1, TFF2, TFF3, and SPDEF in the jejunum of piglets exposed to 1.28 or 2.89 mg DON/kg feed were significantly down-regulated (*P* < 0.05) compared to the control piglets. In the ileum, dietary DON exposure significantly decreased (*P* < 0.05) the mRNA levels of TFF2, TFF3, and SPDEF. In addition, cecal mRNA levels of TFF1, TFF2, TFF3, and SPDEF were lower (*P* < 0.05) in piglets fed the 2.89 mg/kg DON-contaminated diet than those in piglets fed the control diet. However, ingestion of DON had limited effects on the colonic TFFs mRNA expression. We further detected the alteration of Claudin-4 mRNA expression in the intestine. As expected, high level of dietary DON exposure significantly decreased the Claudin-4 mRNA expression in all four different intestinal segments (*P* < 0.05). 

### 2.2. Depression of SPDEF in the Jejunum

**Figure 2:**
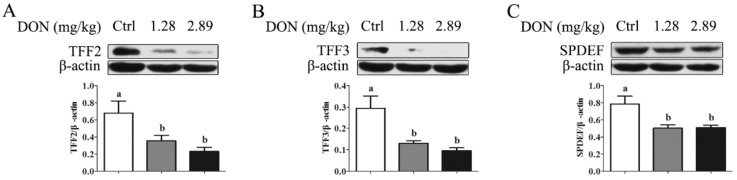
Western blot analysis of the proteins TFF2 (**A**), TFF3 (**B**), and sterile alpha motif (SAM) pointed domain E26 transformation-specific (ETS) factor (SPDEF) (**C**) in the jejunum obtained from piglets fed a control diet (□) or a diet contaminated with 1.28 (■) and 2.89 mg DON/kg feed (■) for 28 days. β-actin was used as a protein loading control. Values are means ± SEM, n = 3. ^a,b^ Mean values without a common letter differ (*P* < 0.05). SEM, standard error of mean.

Western blot results showed that the exposure to 1.28 and 2.89 mg/kg DON for 28 d led to a significant depression (*P* < 0.05) in SPDEF protein abundance, with a consequent decrease (*P* < 0.05) in TFF2 and TFF3 protein level in the jejunum (Figure 2).

### 2.3. TFF Staining in the Jejunum

**Figure 3:**
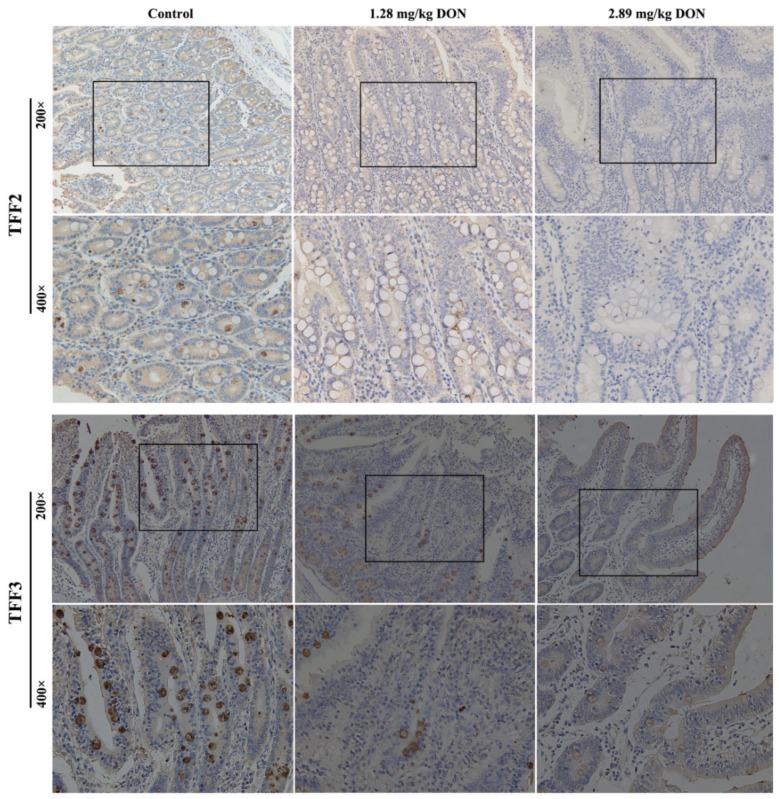
Immune staining of TFF2 and TFF3 in the jejunum of piglets fed a control diet or a diet contaminated with 1.28 and 2.89 mg DON/kg feed. Positive signals are shown by brown color. Magnifications were 200× and 400×. The black squares in the 200× microphotographs exhibit the approximate locations of the 400× microphotographs.

We next investigated tissue localization of TFF2 and TFF3 in the jejunum from different treatments. Immunoreactive TFF2 and TFF3 were readily detected within goblet cells in the jejunum. Notably, dietary exposure to 1.28 and 2.89 mg/kg DON evidently decreased TFF2 and TFF3 expression within goblet cells in the jejunum compared with the control group (Figure 3).

### 2.4. DON Inhibits the mRNA Expression of TFFs by IPEC-J2 cells 

**Figure 4:**
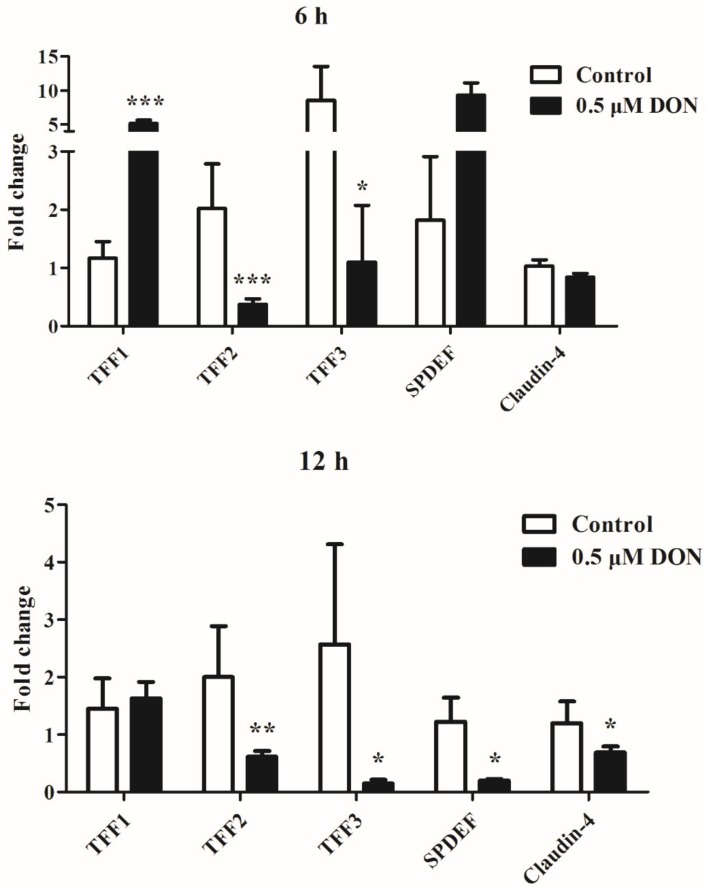
mRNA expression of TFF1, TFF2, TFF3, SPDEF, and Claudin-4 in IPEC-J2 cells with exposure of DON for 6 and 12 h. Values are means ± SEM, n = 6. *P < 0.05, **P < 0.01, ***P < 0.001 compared with the control group. SEM, standard error of mean.

The mRNA expression of TFFs in IPEC-J2 cells after DON exposure was investigated. At 6 h exposure, DON had different effects on TFF1, 2, and 3. DON significantly decreased (*P* < 0.05) the expression of TFF2 and TFF3, whereas it led to a stimulation of TFF1 expression (*P* < 0.001). At 12 h exposure, the mRNA levels of TFF2 and TFF3 were significantly decreased (*P* < 0.05) upon treatment with 0.5 μM DON. Cells exposed to 0.5 μM DON for 12 h had lower (*P* < 0.05) mRNA levels of SPDEF and Claudin-4 compared with the control (Figure 4).

### 2.5. SPDEF was a Critical Regulator Regulating TFFs Expression in Response to DON Exposure

**Figure 5:**
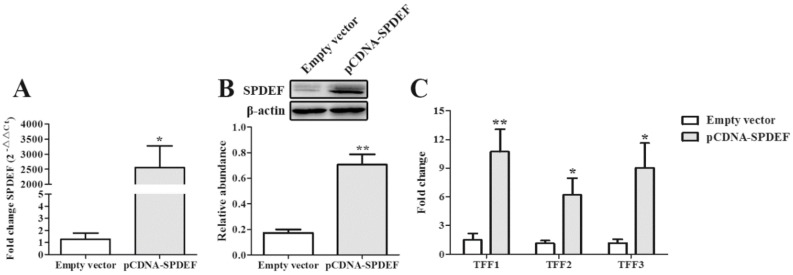
SPDEF regulates the mRNA expression of TFFs in IPEC-J2 cells. qRT-PCR (**A**) and western blot (**B**) showed that SPDEF expression was enhanced in IPEC-J2 cells transfected with pcDNA-SPDEF vector. (**C**) Overexpression of SPEDF increased the mRNA of TFF1, TFF2, and TFF3. An empty vector or SPDEF vector was transfected into IPEC-J2 cells by transient transfection. Values are means ± SEM, n = 3. *P < 0.05 and **P < 0.01 compared with the control group. SEM, standard error of mean.

**Figure 6:**
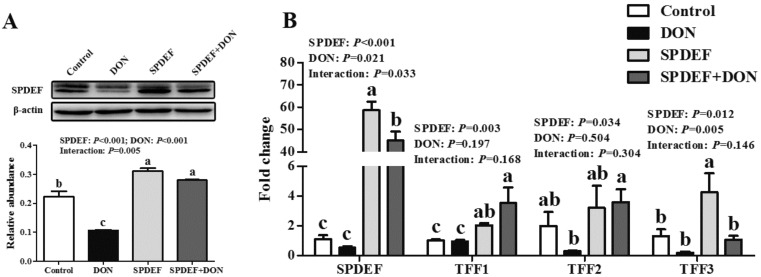
Overexpression of SPDEF attenuated the DON-induced TFFs depression in IPEC-J2 cells. (**A**) Abundance of SPDEF in IPEC-J2 cells transfected with an empty vector or SPDEF vector in the presence or absence of DON. (**B**) mRNA expression of TFFs in IPEC-J2 cells transfected with an empty vector or SPDEF vector in the presence or absence of DON. Values are means ± SEM, n = 3. ^a–c^ Mean values without a common letter differ (*P* < 0.05). SEM, standard error of mean.

To validate the modulatory effect of SPDEF on TFFs expression, IPEC-J2 cells were transfected with an empty vector or SPDEF vector. We confirmed SPDEF overexpression in IPEC-J2 cells by comparing with that in control cells transfected with an empty vector (Figure 5A,B). Overexpression of SPDEF significantly elevated (*P* < 0.05) the mRNA levels of TFF1, TFF2, and TFF3 (Figure 5C). We then examined whether the DON-induced TFFs depression was ameliorated by overexpression of SPDEF. As shown in Figure 6A, DON exposure resulted in a decreased protein level of SPDEF, whereas the inhibitory effect of DON was markedly reversed by the introduction of SPDEF (*P* < 0.01). In the presence of DON, IPEC-J2 cells transfected with SPDEF vector had higher (*P* < 0.05) mRNA levels of SPDEF, TFF1, and TFF2 than those transfected with an empty vector (Figure 6B).

## 3. Discussion

It is well documented that DON negatively affects gut health, such as impairment of intestinal morphology, disruption of barrier function, alteration of nutrient absorption, and gut immunity [8,9,10]. Trefoil factors are bioactive peptides playing crucial roles in mucosal repair and protection within the intestine [14]. Whereas the inhibition effects of DON on TFFs expression by intestinal goblet cells has been reported [4], and its role in modulation of TFFs in piglets is still uncharacterized. We recently showed, using weaned piglets and porcine intestinal epithelial cells (i.e., IPEC-J2 cells), that DON is capable of inhibiting intestinal host defense peptide expression through a nucleotide-binding oligomerization domain protein 2 (NOD2)-dependent activation of caspase-12 [15]. In the current study, the effect of DON on the expression of another indispensable family of peptides, the TFFs, was determined using the same in vivo and in vitro models.

Our results confirmed that dietary DON exposure suppressed the mRNA expression of TFFs in the jejunum, ileum, and cecum of piglets. However, the TFFs mRNA expression in the colon was not significantly affected by the ingestion of DON. We speculated that this could be due to the majority of DON being absorbed in the proximal part of the small intestine. In IPEC-J2 cells, DON inhibited the TFFs mRNA expression in a time-dependent manner, which was consistent with a prior study [4]. In addition, TFF2 and TFF3 in the jejunum were also quantified at the protein level. As expected, we observed an obvious decrease in protein levels of TFF2 and TFF3 in the jejunum of piglets fed 1.28 or 2.89 mg/kg DON-contaminated diet. TFFs are secreted from mucosal epithelium and are mostly synthesized by mucin-producing cells, such as goblet cells [16]. In accordance with previous reports, we demonstrated that immunoreactive TFF2 and TFF3 proteins were present within goblet cells in laminae propria and along the overlying mucosal surface of the jejunum, respectively. In addition, our results showed less TFF2 and TFF3 staining was observed in the jejunum from piglets exposed to a 1.28 or 2.89 mg/kg DON-contaminated diet. A recent in vivo study found that depressed expression of TFF3 was observed in the intestine of mice fed a diet containing a mixture of DON and zearalenone [17]. This result obtained from mice validates our in vivo and in vitro observations, indicating that the effect of DON on TFFs is species-independent, at least in mice and pigs. 

Mice lacking TFFs had an ineffective mucosal repair in response to intestinal injuries by chemicals or radiations [18,19]. It has been reported that TFF3 restores normal intestinal permeability by upregulating tight-junction proteins [20]. In the current study, both the in vivo and in vitro models showed that DON exposure down-regulated the mRNA expression of Claudin-4, which was inconsistent with the findings of Pinton et al. [10]. This effect of DON on tight junction via an inhibition of TFFs expression could not be ruled out.

A recent research demonstrated that the transcription factor SPDEF directly modulates the TFF3 expression in intestinal epithelial cells [12]. We therefore investigated the role of SPDEF in regulating DON-induced depression of TFFs expression. Dietary exposure to 1.28 or 2.89 mg/kg DON decreased SPDEF expression at both mRNA and protein levels. Similar phenomenon was also observed in IPEC-J2 cells. The regulatory relationship was further demonstrated by ectopic expression experiment in IPEC-J2 cells. Intriguingly, cells that stably expressed SPDEF were resistant to DON induced TFFs suppression, indicating a protective effect of SPDEF in response to DON exposure. 

In conclusion, the results of the animal trials showed that dietary DON exposure inhibited the trefoil factors expression in the intestine of piglets. The in vitro study revealed that the DON-induced dysregulation of TFFs was mediated by inhibition of SPDEF, a positive regulator of TFFs. Collectively, these data suggest that the chronic consumption of DON alters intestinal TFFs expression and is alarming. This highlights the DON content in animal feed should be strictly controlled based on the existing regulation for DON from the angle of human health. In addition, our results provide a potential target to prevent the adverse effects of DON. 

## 4. Materials and Methods

### 4.1. Animals and Experimental Design

All animal experimental procedures were performed according to the Institutional Animal Care and Use Committee of Huazhong Agricultural University (Wuhan, China). The project approval code is HZAURA-2015-006. Twenty-four 28-d-old castrated male piglets (Duroc × Landrace × Large White; initial body weight = 7.6 ± 0.7 kg) were randomly divided into three dietary groups: control diet (0.61 mg DON/kg feed) and two diets contaminated with 1.28 and 2.89 mg DON/kg feed, respectively. Diets were formulated to meet the nutrient requirements of swine as recommended by the National Research Council [21]. The DON-contaminated feed was prepared as previously described [15]. Piglets were individually housed in metabolic cages and allowed free access to water and feed during the 28 day experiment period. The formulation and nutrient levels of the basal diet has been presented by Wang et al. [15]. 

On day 28, all piglets were euthanized and samples were collected to detect the intestinal TFFs expression. Middle sections (3 cm) of the jejunum, ileum, cecum, and colon were carefully removed and gently flushed with 0.9% physiological saline. Then the intestinal segments were immersed in liquid nitrogen and stored at −80 °C for real-time PCR. These mid-jejunum samples were also used for western blot analysis. In addition, 3 cm of the mid-jejunum was isolated and fixed in 4% paraformaldehyde for subsequent immunohistochemistry staining.

### 4.2. Immunohistochemistry 

Jejunal sections (5 μm) embedded in paraffin were incubated with primary antibodies for TFF2 and TFF3 (ABclonal, Wuhan, China) at 4 °C overnight followed by incubation with an anti-rabbit biotinylated secondary antibody. Specific binding was detected using a peroxidase-conjugated streptavidin assay kit (Sigma Chemical Co., St Louis, MO, USA). The slides were counterstained with hematoxylin. Images were captured on an Olympus BX53 microscope equipped with an Olympus DP73 camera (Olympus, Tokyo, Japan). 

### 4.3. Cell Culture and Treatment

The IPEC-J2 cell is a non-transformed epithelial cell line separated from the jejunum of a newborn pig. This cell line maintains most of its epithelial nature. IPEC-J2 cells were grown in DMEM-F12 medium supplemented with 10% FBS and 100 units/mL penicillin/streptomycin at 37 °C in a humidified 5% CO_2_ atmosphere. The culture was changed every other day.

For stimulation experiments, IPEC-J2 cells were incubated overnight in 6-well plates at a density of 1 × 10^6^ cells per well. After reaching 80% confluence, cells were exposed to 0.5 μM DON (Sigma Aldrich, St. Louis, MO, USA) for 6 or 12 h. At the indicated time, total RNA from IPEC-J2 cells were extracted to detect the mRNA expression of TFFs.

### 4.4. Plasmid Construct and Transfection

To construct pcDNA-SPDEF, a DNA fragment containing the porcine SPDEF coding sequences was generated by PCR amplification and cloned into BamHI and EcoRI-restricted pcDNA 3.1 (+) by a commercial company (GenePharma, Shanghai, China). This construct was verified by DNA sequencing. Transient transfection of plasmid into IPEC-J2 cells was performed using lipofectamine 2000 (Invitrogen, Carlsbad, CA, USA) following the manufactures protocol. 

After incubation for 24 h, transfected cells were treated with or without 0.5 μM DON for another 12 h. Then, total RNA and protein were extracted for quantitative PCR and immunoblotting, respectively.

### 4.5. Quantitative PCR

Total RNA from the intestinal tissues and IPEC-J2 cells was extracted using TRIzol (Takara, Dalian, China), and quantified by a NanoDrop 2000 (Thermo Fisher Scientific, Waltham, MA, USA). One microgram RNA was reverse transcribed to cDNA with an ABScript II cDNA First Strand Synthesis Kit (ABclonal, Wuhan, China). Primers for TFFs (TFF1, TFF2, and TFF3), SPDEF, Claudin-4, and β-actin are given in Table 1. The selected genes were detected on a Bio-Rad CFX384 Real-Time PCR System with SYBR Green Fast qPCR Mix (ABclonal, Wuhan, China). The relative mRNA expression of these genes was normalized with β-actin using 2^−ΔΔCt^ formula [22,23]. 

### 4.6. Western Blotting

Western blot analysis of jejunum tissues or IPEC-J2 cells was performed as previously described [15]. Primary antibodies against TFF2, TFF3, and SPDEF (ABclonal, Wuhan, China) were used. Blots were stripped and reprobed with anti-β-actin antibody (ABclonal, Wuhan, China) to demonstrate equal loading.

### 4.7. Statistical Analysis

Data of in vivo trial were analyzed by one-way ANOVA with a Bonferroni post hoc test. For the in vitro experiments, differences between groups were determined using unpaired Student’s two-tailed t-test. In addition, two-factor ANOVA was used to compare the effects of SPDEF overexpression with and without DON treatment. Data were expressed as mean ± standard error of mean (SEM). Differences were considered significant when *P* value was less than 0.05. Analyses were performed by using SAS (version 9.2, SAS Institute, Inc., Gary, NC, USA).

## Figures and Tables

**Table 1:** Sequence of the primers used for RT-qPCR.

Gene	Forward Primer (5′–3′)	Reverse Primer (5′–3′)	Accession No.
*β-actin*	TACACCGCTACCAGTTCGC	GCTCGATGGGGTACTTGAGG	XM_021086047
*TFF1*	TGCCAGAGTGAACTGTGGTTTC	CAAAGCAGCAGCCTTTTTTTTC	AM283538.1
*TFF2*	ATCACCAGCGACCAGTGCTT	ATGACGCACTCCTCAGACTCTTG	XM_003358971.1
*TFF3*	CAGGATGTTCTGGCTGCTAGTG	GCAGTCCACCCTGTCCTTG	NM_001243483.1
*SPDEF*	CATTCACCTGTGGCAGTTCC	TAGTTCATGGCAGGACGGTT	NM_001190254
*Claudin-4*	CTCTCGGACACCTTCCCAAG	GCAGTGGGAAGGTCAAAGG	NC_010445.4
